# Construction and Analysis of Disuse Atrophy Model of the Gastrocnemius Muscle in Chicken

**DOI:** 10.3390/ijms23136892

**Published:** 2022-06-21

**Authors:** Jiawei Mo, Zhijun Wang, Qingchun Liu, Zhenhui Li, Qinghua Nie

**Affiliations:** 1 State Key Laboratory for Conservation and Utilization of Subtropical Agro-Bioresources & Lingnan Guangdong Laboratory of Agriculture, College of Animal Science, South China Agricultural University, Guangzhou 510642, China; jiawei-mo@stu.scau.edu.cn (J.M.); zhijunwang@stu.scau.edu.cn (Z.W.); 2774844295@scau.edu.cn (Q.L.); lizhenhui@scau.edu.cn (Z.L.); 2 Guangdong Provincial Key Lab of Agro-Animal Genomics and Molecular Breeding and Key Laboratory of Chicken Genetics, Breeding and Reproduction, Ministry of Agriculture, Guangzhou 510642, China; 3 National-Local Joint Engineering Research Center for Livestock Breeding, Guangzhou 510642, China

**Keywords:** chicken, RNA-seq, disuse muscle atrophy, oxidative stress, ROS

## Abstract

Disuse muscle atrophy is identified as the physiological, biochemical, morphological, and functional changes during restricted movement, immobilization, or weightlessness. Although its internal mechanism has been extensively studied in mammals and was thought to be mainly related to oxidative stress, it was unclear whether it behaved consistently in non-mammals such as chickens. In this study, we tried to construct a disuse atrophy model of the gastrocnemius muscle in chickens by limb immobilization, and collected the gastrocnemius muscles of the fixed group and the control group for RNA sequencing. Through analysis of muscle loss, HE staining, immunohistochemistry, and oxidative stress level, we found that limb immobilization could lead to loss of muscle mass, decrease in muscle fiber diameter, decrease in the proportion of slow muscle fibers, and increase in the proportion of fast muscle fibers, and also cause elevated levels of oxidative stress. In addition, a total of 565 different expression genes (DEGs) were obtained by RNA sequencing, which was significantly enriched in the biological processes such as cell proliferation and apoptosis, reactive oxygen species metabolism, and fast and slow muscle fiber transformation, and it showed that the FOXO signaling pathway, closely related to muscle atrophy, was activated. In brief, we initially confirmed that limb immobilization could induce disuse atrophy of skeletal muscle, and oxidative stress was involved in the process of disuse muscle atrophy.

## 1. Introduction

Skeletal muscle is the heaviest tissue in the animal body, accounting for about 40% of the total body of the animal [1]. It plays an important role in the normal growth and development of the animal, metabolism, movement, and brake [2]. In addition, the skeletal muscle has a high degree of plasticity, and its physiological characteristics are also highly variable. In the face of potential external stimuli, skeletal muscle can undergo extensive reconstruction to meet the needs of contraction and energy metabolism [3,4].

Disuse muscle atrophy is a nonpathological symptom of a series of physiological, biochemical, morphological, and functional changes that occur in the state of hypokinesia, immobilization (fixation), or weightlessness of limb muscles. It is manifested in the microstructure that muscle protein anabolism is weakened, catabolism is enhanced, and glycogen intake and synthesis rate are decreased. Additionally, in the macrostructure, it is manifested as a decrease in muscle mass, a decrease in muscle fiber cross-sectional area, a muscle fiber transformation from a slow type (type I) to a rapid type (type II), and so on [5,6]. It has been found in clinical medicine that working in a weightless environment for a long time [7], as well as some diseases (such as hemiplegia caused by stroke [8]) or treatment (such as cast immobilization after fracture [9]) accompanied by hypokinesia or forced immobilization, will lead to disuse muscle atrophy.

Oxidative stress (OS) means that when the body is damaged or subjected to various harmful stimuli, the rate of generation of oxidative free radicals is greater than the rate of elimination and out of balance, showing a state of oxidative damage [10,11]. The former refers to the highly active molecules produced by organisms in the metabolic process, such as reactive oxygen species (ROS) and reactive nitrogen species (RNS); the latter mainly refers to enzyme antioxidant systems, such as superoxide dismutase (SOD), catalase (CAT), and glutathione peroxidase (GSH-Px), and nonenzymatic antioxidant systems composed of vitamin E, glutathione, and carotenoids. Studies have shown that in the artificially simulated muscle atrophy model, the process of disuse muscle atrophy is accompanied by a certain degree of oxidative stress. For example, the enzymatic and nonenzymatic antioxidant capacity of the soleus muscle in a rat model of muscle atrophy caused by hindlimb unloading is significantly reduced, and the level of lipid peroxidation in that is significantly increased [12,13]. Additionally, in a mouse model of denervation-induced muscle atrophy, the reactive oxygen species (ROS) products of gastrocnemius mitochondria increase significantly [14]. There is increasing evidence that oxidative stress plays an important role in the occurrence of muscle wasting [15,16,17].

In this study, we constructed a broiler disuse muscle atrophy model based on the method of limb immobilization and then identified 565 DEGs through RNA sequencing. Moreover, we conducted a series of experiments to explore the various changes in skeletal muscle during disuse atrophy, including fiber morphology, antioxidant level, and gene expression level. In general, we successfully constructed a disuse atrophy model of the gastrocnemius muscle in chicken, and tried to explore the molecular mechanism of disuse muscular atrophy from the level of gene expression.

## 2. Results

### 2.1. Atrophy Model Leads to Loss of Muscle Mass

**Figure 1:**
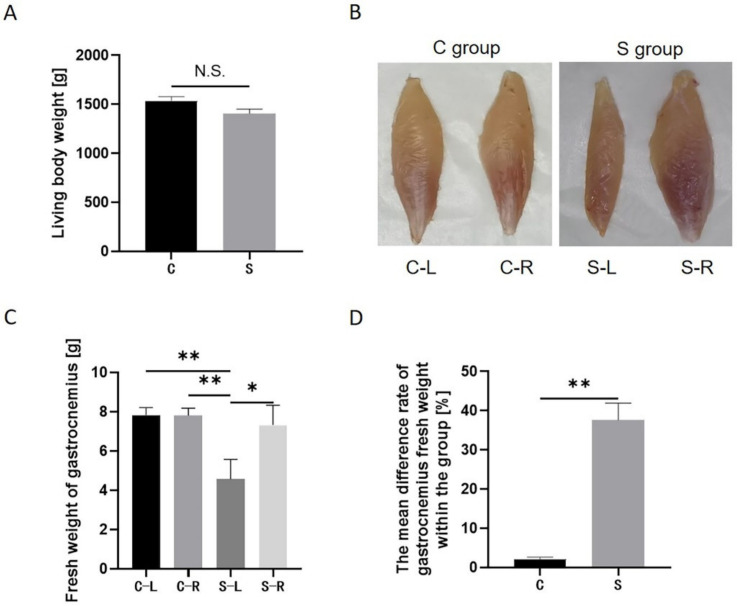
Atrophy model leads to loss of muscle mass. (**A**) Live body weight of each group before sampling. (**B**) Schematic diagram of the gastrocnemius muscle of each group after sampling. (**C**) Fresh weight of gastrocnemius muscle of each group. (**D**) Mean difference rate of gastrocnemius fresh weight within the group. Data are presented as mean ± SEM (*n* = 6 biologically independent samples). * *p* < 0.05; ** *p* < 0.01 (Student’s *t*-test).

Under the treatment of unilateral limb fixation, the gastrocnemius of the S-L group (see Table 1 for details) showed a significant change. In the case of similar living body weights between the treatment group and the control group (Figure 1A), the fresh weight of the gastrocnemius in the S-L group was significantly smaller than that in the S-R group (see Table 1 for details) and the control group (including the C-L group and C-R group, see Table 1 for details). The fresh weight of the gastrocnemius in the S-L group was reduced by an average of 37.59% compared to the S-R group, and by an average of 41.47% compared to the control group (including the C-L group and C-R group) (Figure 1C). In addition, there was no significant difference in the fresh weight of the gastrocnemius among the C-L group, C-R group, and S-R group (Figure 1C). In addition, the mean difference rate of gastrocnemius fresh weight within the S group (see Table 1 for details) was significantly bigger than that within the C group (see Table 1 for details) (Figure 1D). This suggests that external fixation of the limb leads to loss of muscle mass.

### 2.2. Atrophy Model Results in a Reduction of Muscle Fiber Diameter and Altered Muscle Fiber Composition Type

**Figure 2:**
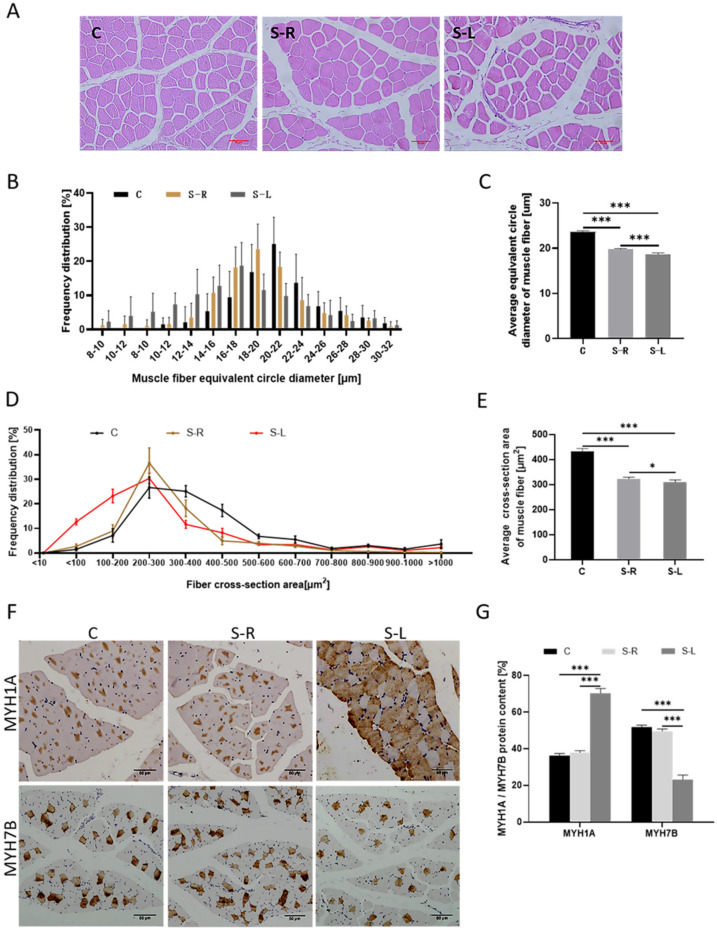
Atrophy model results in reduction of muscle fiber diameter and altered muscle fiber composition type. (**A**) Hematoxylin and eosin (H&E) staining of gastrocnemius muscle (scale bar = 50 μm). (**B**) Distribution of muscle fiber diameter size. (**C**) Statistics of average muscle fiber diameter size. (**D**) Distribution of cross-sectional area of muscle fiber. (**E**) Statistics of average cross-sectional area of muscle fiber. (**F**) Immunohistochemical staining for composition type of gastrocnemius muscle fiber. (**G**) Statistics of MYH1A/MYH7B protein content for composition type of gastrocnemius muscle fiber. Data are presented as mean ± SEM (*n* = 3 biologically independent samples). * *p* < 0.05; *** *p* < 0.001 (Student’s *t*-test).

The changes in muscle fibers’ diameter were observed by HE staining. The results showed that the area of muscle bundles in the S-L group was smaller than that in the S-R group and the C group, and the muscle bundle space was larger than in the S-R group and the C group (Figure 2A,B). The average diameter of muscle fibers of the S-L group is 18.68 ± 6.68 μm, which is very significantly smaller than 19.74 ± 4.55 μm of the S-R group and 22.65 ± 6.17 μm of the C group (Figure 2C). Additionally, the diameter of the muscle fibers presents an approximately normal distribution. Moreover, the distribution of the muscle fiber cross-sectional area also showed the same trend as that of the muscle fiber diameter (Figure 2D). The average cross-sectional area of muscle fibers of the S-L group is 308.84 ± 9.90 μm^2^, which is significantly smaller than 322.21 ± 6.73 μm^2^ of the S-R group and 432.63 ± 11.36 μm^2^ of the C group (Figure 2E).

In addition, the changes in muscle fibers’ composition types were observed by immunohistochemical staining. The results showed that the content of the fast muscle fiber subtype MYH1A in the S-L group was extremely significantly higher than that of the S-R group. The content of the slow muscle fiber subtype MYH7B in the S-L group was extremely significantly lower than that of the S-R group (Figure 2F,G), and it presented the same result compared with the C group. This indicates that the muscle fiber may transform from slow type (type I) to fast type (type II) under disuse atrophy, resulting in a decrease in slow muscle fibers and an increase in fast muscle fibers.

### 2.3. Increased Levels of Oxidative Stress in Atrophy Model

**Figure 3:**
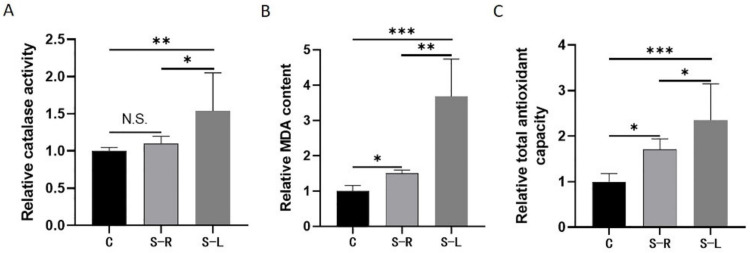
Increased levels of oxidative stress in atrophy model. (**A**) Relative catalase activity analysis. (**B**) Relative MDA content analysis. (**C**) Relative total antioxidant capacity analysis. Data are presented as mean ± SEM (*n* = 3 biologically independent samples). * *p* < 0.05; ** *p* < 0.01; *** *p* < 0.001 (Student’s *t*-test).

In order to explore the oxidative stress response of unilateral limb fixation, we detected catalase (CAT) activity, lipid peroxide (MDA) content, and total antioxidant capacity in the gastrocnemius muscle samples. The results showed that, compared to the C group, the CAT activity of the S-L group was significantly increased by about 0.5 times, the MDA content was significantly increased by about 1.5 times, and the total antioxidant capacity was significantly increased by about 1 time; and compared to the S-R group, the S-L group showed the same change trend as before (Figure 3). Surprisingly, the S-R group has an upward trend in its CAT activity, MDA content, and total antioxidant capacity compared with the C group. The rise of these biochemical indicators indicates that a quite strong oxidative stress occurs in the process of disuse muscle atrophy, leading to a significant increase in peroxidation products. At the same time, the organism will spontaneously upregulate the function of its antioxidant system to maintain relatively healthy homeostasis.

### 2.4. Transcriptome Sequencing Analysis

According to the degree of gastrocnemius atrophy, we selected 4 individuals from the S group, 3 individuals from the C group, and a total of 14 gastrocnemius samples for transcriptome sequencing, which were divided into 3 groups, including the C group, S-R group, and S-L group. Additionally, we obtained 7.52 Gb of raw data for each sample on average (Table S1), which were submitted to the NCBI database (Accession Number: PRJNA777906). After filtrating the raw reads, 6.85 Gb of total clean reads were obtained. The average GC content reached 50.50%, and the base proportion reaching the Q30 standard was no less than 87.47%. The clean reads were mapped to the reference genome (GRCg6a), the average mapping ratio was 85.19%, and the unique mapping ratio was not less than 80.73% (Table S2).

### 2.5. DEGs among Three Groups (C Group, S-R Group, and S-L Group) of Gastrocnemius Tissues

**Figure 4:**
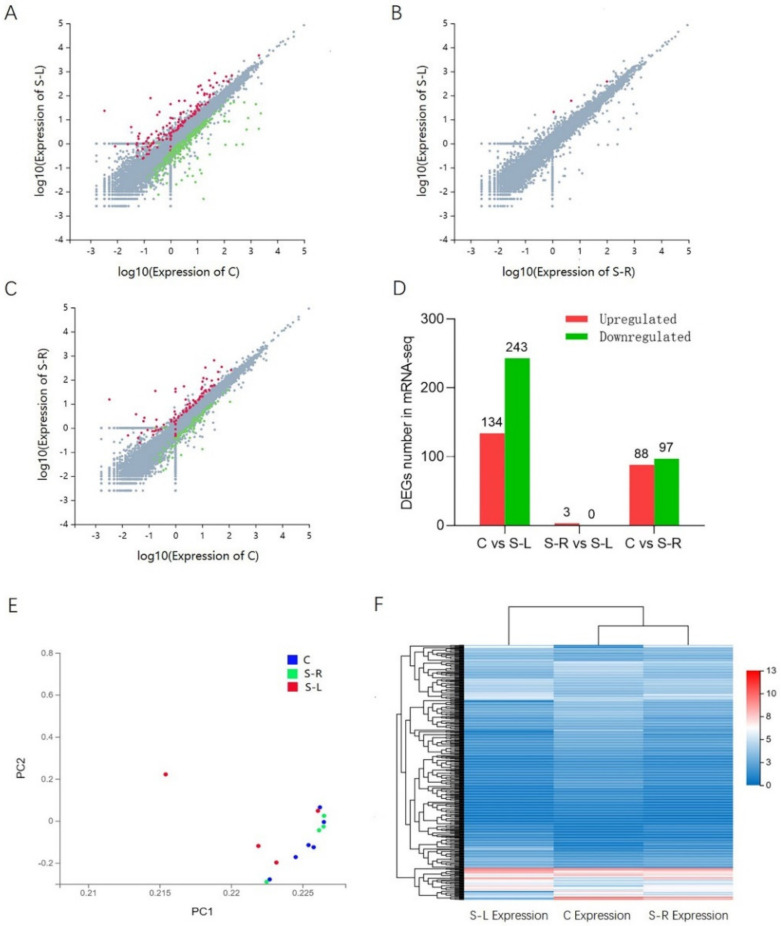
DEGs among 3 groups of gastrocnemius tissues. (**A**–**C**) Scatter plots of mRNA expression distribution between S−L group and C group, or between S−L group and S−R group, or between S−L group and C group. Color: Blue indicates downregulated genes, red indicates upregulated genes, and gray indicates nondifferentially expressed genes. (**D**) Quantity statistics of DEGs, in which the red column represents upregulated DEGs and the green represents downregulated DEGs. (**E**) Principal component analysis of differentially expressed genes among 3 groups. (**F**) Heatmap of differentially expressed genes (DEGs) among 3 groups, in which rows represent genes, and columns represent different groups.

To identify the potential candidate genes related to disuse muscle atrophy, the expression levels of mRNA were examined in gastrocnemius tissues from the three groups. Q-value ≤ 0.05 and |fold change| ≥ 1 were set as the standard for differential expression. On the one hand, a total of 16,222 genes were detected, and a total of 565 differentially expressed genes were identified in a pairwise comparison between the three groups. Among them, compared with the C group, it showed that 134 genes were upregulated, and 243 genes were downregulated in the S-L group; however, compared with the S-R group, it showed that only three genes were upregulated in the S-L group. Moreover, compared with the C group, 88 genes were upregulated and 97 genes were downregulated in the S-R group (Figure 4A–D). On the other hand, both the dimensionality reduction analysis of DEGs in PCA (Figure 4E) and the clustering analysis of DEGs in the heat map (Figure 4F) showed that the gene expression patterns of the S-R group and the C group were much similar, and the gene expression patterns of the S-L group were quite different from those of the two groups. This indicates that unilateral limb fixation is successful, and it can specifically induce disuse muscle atrophy in the limbs, which in turn triggers differences in gene expression patterns. Additionally, after considering the experimental purpose of exploring disuse muscle atrophy and the feasibility of data analysis, we focused on the differences in gene expression patterns between the S-L group and the C group, not those between the S-R group and the C group, nor those between the S-L group and the S-R group.

### 2.6. GO and KEGG Pathway Analysis for DEGs

**Figure 5:**
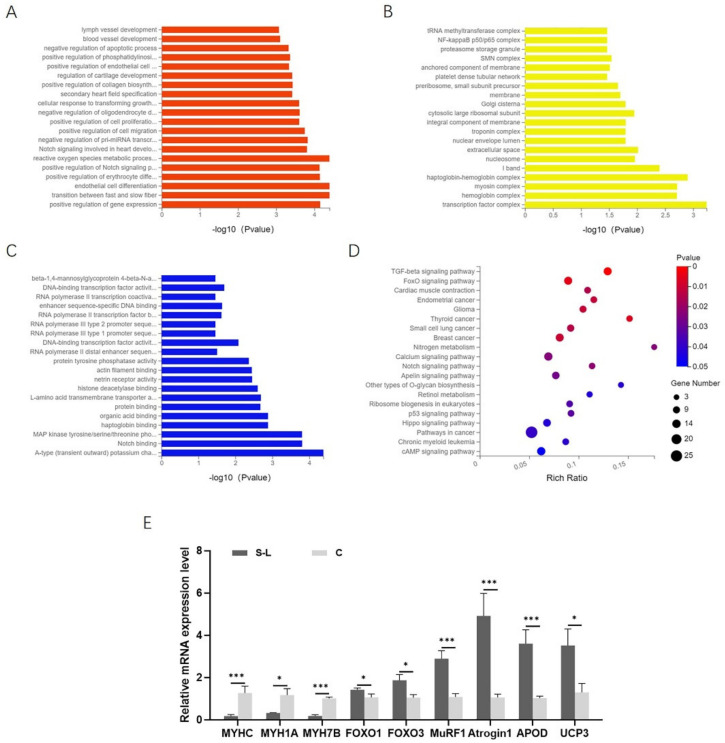
GO and KEGG pathway analysis for DEGs. (**A**) Top 20 terms of the biological process part of GO enrichment analysis for DEGs. (**B**) Top 20 terms of cell component part of GO enrichment analysis for DEGs. (**C**) Top 20 terms of molecular functions of GO enrichment analysis for DEG. (**D**) Top 20 enriched pathways in KEGG pathway analysis for DEGs. (**E**) Validation of transcriptome sequencing results by qPCR. Data are presented as mean ± SEM (*n* = 6 biologically independent samples). * *p* < 0.05; *** *p* < 0.001 (Student’s *t*-test).

To explore the involvement of DEGs in disuse muscle atrophy, GO enrichment analysis and KEGG pathway analysis were performed between the S-L group and the C group. The GO enrichment analysis was divided into three parts based on functional annotations, including biological processes (BPs), cell components (CCs), and molecular functions (MFs). Additionally, a value of *p* < 0.05 was considered as the criterion for significant enrichment. The DEGs could be classified in 1762 terms for BPs, 247 terms for CCs, and 483 terms for MFs. Among them, 470 terms, 26 terms, and 80 terms were significantly enriched, respectively (Table S3). Some terms that may be related to disuse muscle atrophy were enriched, including the process of reactive oxygen metabolism, the transition between fast and slow muscle fibers, the positive regulation of phosphatidylinositol 3-kinase signaling, the development of blood vessels, and the negative regulation of apoptosis (Figure 5A), myosin complex, troponin complex (Figure 5B), and MAP kinase activity (Figure 5C).

In the KEGG pathway analysis, the results showed that DEGs were enriched in 256 pathways. A value of *p* ≤ 0.05 was regarded as a significant difference in statistics of pathway enrichment. The top 20 enrichment pathways are shown in Figure 5D and listed in Table 2. Because disuse muscle atrophy mainly manifested as decreased muscle mass and active oxidative stress response, we paid more attention to the following signaling pathways, including the calcium signaling pathway related to muscle contraction, the p53 signaling pathway, and TGF-β signaling pathways related to cell proliferation and differentiation, FOXO signaling pathways related to antioxidative stress, cAMP signaling pathways, and PI3K-AKT signaling pathways involved in intracellular and extracellular signal transmission. The differential expression of related genes in these signaling pathways makes the muscles maintain an atrophy status.

Furthermore, we also found that in the S-L group, genes related to the ATP–ubiquitin–proteasome proteolytic pathway (*FOXO3*, *MuRF1*, *Atrogin1*, *UBAP2*, *UBB*, *UBC*, *UBE2R2L*) were significantly upregulated, genes related to skeletal muscle slowed muscle fiber types (*MYH7B*, *TNNC1, TNNI1*, *TNNT1*) were significantly downregulated, and genes associated with ROS metabolism (*ApoD*, *UCP3*, *SOD1*, *SESN1*, *PDK4*, *AIFM2*) were significantly upregulated (Table S4). It is preliminarily indicated that the protein catabolism in disuse muscle atrophy is mainly carried out through the ATP–ubiquitin–proteasome proteolytic pathway, and ROS may be involved in the process of regulating muscle atrophy.

### 2.7. The ROS Can Induce Myotube Atrophy

In order to prove that ROS could cause disuse muscle atrophy, we used the chicken primary myoblasts (CPMs) for verification. The CPMs were isolated from the leg muscles of 11 embryonic chicken embryos and next cultured to differentiate and fuse into the myotube. Finally, we added 2,3-dimethoxy-1,4-naphthoquinone (DMNQ) at a concentration of 30 μmol/L to initiate an intracellular ROS reaction, which was used in the following corresponding experiments.

**Figure 6:**
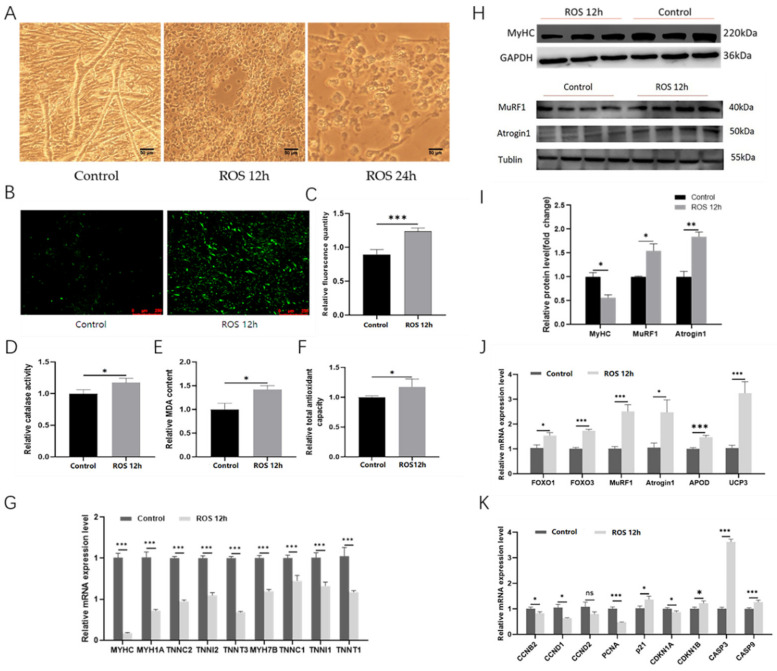
ROS induces myotube atrophy. (**A**) Morphological changes in CPM treated with DMNQ for 12 h and 24 h. (**B**) Fluorescence map for ROS level in CPM treated with DMNQ for 12 h. (**C**) Statistics of the fluorescence quantity for ROS content in CPM treated with DMNQ for 12 h. (**D**) Analysis of CAT activity in CPM under DMNQ treatment for 12 h. (**E**) Analysis of MDA content in CPM under DMNQ treatment for 12 h. (**F**) Analysis of total antioxidant capacity in CPM under DMNQ treatment for 12 h. (**G**) Expression of genes related to muscle fiber types after treatment with DMNQ for 12 h. (**H**) Grayscale graph of the protein expression level after treatment with DMNQ for 12 h. (**I**) Statistical graph of the protein expression level after treatment with DMNQ for 12 h. (**J**) Expression of genes related to muscle atrophy and potential anti-oxidation after treatment with DMNQ for 12 h. (**K**) Expression of genes related to cell cycle and apoptosis after treatment with DMNQ for 12 h. Data are presented as mean ± SEM (*n* = 6 biologically independent samples). * *p* < 0.05; ** *p* < 0.01; *** *p* < 0.001 (Student’s *t*-test).

The experimental results showed that after 12 h of DMNQ treatment, the myotube morphology changed from a long fibrous shape to obvious collapse and atrophy, and after treatment for 24 h, most of the CPM had died (Figure 6A). Under DMNQ treatment for 12 h, the level of ROS in the cell was increased significantly (Figure 6B,C); moreover, the CAT activity, MDA content, and total antioxidant capacity were enhanced (Figure 6D–F). Additionally, we also found that the intracellular ROS reaction could downregulate the mRNA expression of *MyHC*, *MYH1A*, and *MYH7B*. (Figure 6G); similarly, the expression of the MyHC protein was proved to decrease by the Western blot (Figure 6H,I), which means that myosin was losing, and the myotube was atrophying. The mRNA expression of the key genes to skeletal muscle atrophy, such as *FOXO1*, *FOXO3*, *MuRF1*, and *Atrogin1*, was upregulated significantly (Figure 6J), being in accord with our expectations. Moreover, the expression of MuRF1 and Atrogin1 protein was also proved to increase by the Western blot (Figure 6H,I). The mRNA expression of genes related to cell cycle and apoptosis indicates that the ROS could inhibit cell proliferation by cell cycle arrest and promote apoptosis (Figure 6K). Surprisingly, we also found the mRNA expression of genes associated with potential antioxidation, such as *ApoD* and *UCP3*, was upregulated sharply under the action of ROS, which was consistent with that in RNA-seq. In short, this shows that the ROS could induce myotube atrophy, the changes in the antioxidant index, and the mRNA expression of genes, such as that in disuse muscle atrophy.

## 3. Discussion

Disuse muscle atrophy is common in daily life. It often occurs in humans when they have been staying in bed for a long time or taking plaster fixation measures for fractures [18]. Additionally, in animals, it occurs when the amount of exercise is reduced objectively due to limited space for movement under the intensive production of animal husbandry, which results in a certain degree of disuse muscle atrophy. In our study, we used 49-day-old Sanhuang chickens as the experimental object, built a broiler disuse muscle atrophy model by limb immobilization, and then explored the changes in fiber morphology, antioxidant level, and expression of DEGs. Through RNA sequencing, it was found that in the process of disuse muscle atrophy, the p53 signaling pathway, related to cell proliferation and apoptosis, and the FOXO signaling pathway, related to intracellular protein metabolism, are promoted, and the intracellular Ca+ metabolic pathway is inhibited.

It was reported that after 4 weeks of immobilization, the wet weights of gastrocnemius muscle were lost, and the cross-sectional area of gastrocnemius muscle fibers was decreased in rabbits [19]. The cross-sectional area of gastrocnemius muscle fibers, especially type I muscle fibers, was also significantly decreased in mice under hindlimb suspension for 2 weeks [20,21]. Additionally, after unloading the hindlimb muscles by hanging, the expression of the type I MyHC subtype of the antigravity muscles was significantly reduced, but the expression of the type II MyHC subtype was increased [22], and the level of oxidative stress was also increased [23]. This is very similar to our findings on the changes in muscle fiber morphology and antioxidant levels in a broiler disuse muscle atrophy model, but the specific regulatory mechanism needs to be studied further. Additionally, in muscle atrophy caused by immobilization, slow muscle fibers lose more, which may be related to the different metabolic methods of different types of muscle fibers. Slow-twitch fibers are mainly supplied by aerobic oxidation, and fast-twitch fibers are mainly supplied by glycolysis. Under the circumstances of oxidative stress, the function of intracellular mitochondria will be inhibited or even damaged, affecting aerobic oxidative energy supply [24,25], which makes slow-twitch muscle fibers more prone to atrophy. This is bad news for the broiler production industry, because some studies have shown that meat production is mainly related to the quality of skeletal muscle, and the muscle quality of broilers is importantly related to the composition of muscle fiber types [26]. Additionally, it is generally believed that the quality of muscles rich in slow-type (type I) muscle fibers is better for the reason that they have a small diameter, rich myoglobin, low glycogen content, and high intramuscular lipid content, and their tenderness, flesh color, and flavor are better [27]. However, under the modern breeding model, broiler chickens are mainly raised in dense cages, and their activity has dropped sharply. In fact, a disused muscle atrophy model caused by hypokinesis has formed, which may affect the meat production and muscle quality of broilers to a certain extent.

In addition, we found an intriguing result that only three genes were upregulated but not downregulated between the S-L group and the S-R group, along with the fact that the level of muscle atrophy in the S-L group was evident. For this, we speculated that the samples of S-L and S-R were from the same individual, and the increased oxidative stress level caused by limb immobilization affected the whole body through the blood circulation system, resulting in fewer differences in gene transcriptional level. Similar situations were reported in ovarian cancer disease in women, pseudoxanthoma elasticum (PXE) patients, and some postoperative complications, which all showed that oxidative stress could affect the body through the blood circulation system [28,29,30].

With the progress of research, there is increasing evidence that oxidative stress plays an important role in the occurrence of muscle atrophy [31,32,33]. It has been reported that ROS can activate the NF-κB and FOXO signaling pathways (such as *FOXO1* and *FOXO3*) in disuse muscle atrophy and then significantly upregulate the expression of *MuRF1* and *Atrogin1* [34,35]. Studies have shown that MuRF1 and Atrogin1 are key functional molecules in the ubiquitin–proteasome pathway, which is the major pathway involved in protein degradation in skeletal muscle cells [36]. Additionally, almost all cases of muscle atrophy have been accompanied by upregulated expression of *MuRF1* and *Atrogin1* [37]. MuRF1 can bind and degrade myosin heavy-chain (MyHC) proteins after treatment of skeletal muscle with dexamethasone [38]. Moreover, overexpression of *FOXO3* can promote transcription of *MuRF1* and *Atrogin1* and then activate the ATP–ubiquitin–proteasome pathway and autophagy/lysosome pathway to induce myotube atrophy in C2C12 cells [39]. In our experimental data, after DMNQ treatment, on the one hand, the cells showed a trend of shrinking, rounding, and falling off morphologically; on the other hand, the qPCR results showed that *FOXO1*, *FOXO3*, *MuRF1*, and *Atrogin1* were significantly upregulated, and the Western blot results showed that the protein expression of MuRF1 and Atrogin1 was increased, and the protein expression of MyHC was decreased. In short, we believe that DMNQ acted on cells to generate ROS and then directly or indirectly upregulated the expression of MuRF1 and Atrogin1, which promoted the degradation of proteins such as MyHC, and ultimately led to myotube atrophy.

Additionally, it has been suggested that the formation of reactive aldehydes (such as 4-hydroxy-2,3-trans-nonenal) mediated by oxidants could reduce the activity of Ca^2+^-ATPase on the plasma membrane and affect intracellular calcium ion homeostasis [40]. Increasing intracellular Ca2+ concentration could specifically activate calpain, which can participate in protein degradation mediated by the proteasome system [41,42,43]. Additionally, calpain can dissociate the intact myosin complex into monomeric contractile proteins (actin and myosin). These proteins can be degraded by the proteasome system, which is the rate-limiting step of muscle protein degradation [44]. Interestingly, it has also been shown that the addition of the sarcoplasmic reticulum (SR) Ca^2+^ ATPase (*SERCA*) activator *CDN1163* ameliorates muscle oxidative stress injury induced by Cu-Zn-SOD deficiency (Sod1−/−) in mice [45]. It was consistent with our transcriptome sequencing data that genes related to encoding membrane Ca^2+^-ATPase were significantly downregulated, including *ATP2A2*, *ATP2A3*, and *ATP2B2*, when muscle atrophy occurred.

In addition, we also found that *ApoD* and *UCP3*, related to ROS metabolism, are highly expressed. Additionally, it is beneficial for the body’s cells to alleviate and resist the oxidative stress caused by ROS so as to maintain the body’s health and homeostasis. It has been reported that when inducing oxidative stress by paraquat (PQ), *ApoD* plays a protective role in biological survival by regulating tissue homeostasis and maintaining low levels of lipid peroxidation [46]. *ApoD* helps to reduce the level of inflammation in ROS-damaged tissues and promotes the clearance of lipid peroxides in cells [47]. Moreover, uncoupling proteins (UCPs) can participate in intracellular antioxidant defense in a gentle uncoupling manner [48,49]. Overexpression of *UCP3* can inhibit the production of ROS [50,51]. Compared with wild-type mice, *UCP3* knockout mice can produce more ROS in skeletal muscle mitochondria [52]. Additionally, lipid peroxidation products can also activate UCPs to increase proton leakage of the inner mitochondrial membrane, which inhibits the massive generation of ROS with a negative feedback method of gentle uncoupling [53].

To date, the extrinsic causes of disuse muscle atrophy have mostly been attributed to exercise restriction (including external fixation and prolonged bed rest) and a weightless environment, but the underlying molecular mechanism remains unclear. With the progress of research, there is increasing evidence that severe oxidative stress plays an important role in disuse muscle atrophy. The excessive production of ROS is likely to be the cause of muscle atrophy, but there is no direct evidence. In summary, our research also proved that external fixation can induce disuse muscle atrophy in the gastrocnemius muscle of chickens, and found that the level of oxidative stress in disuse muscle atrophy tissue is substantially elevated, which provides evidence for oxidative stress leading to disuse muscle atrophy. Moreover, we also found two potential regulatory genes associated with disuse muscle atrophy, *ApoD* and *UCP3*.

## 4. Materials and Methods

### 4.1. Ethics Statement

The Institutional Animal Protection and Utilization Committee of South China Agricultural University approved the animal experiments conducted in this study. The experiment was carried out following established regulations and guidelines. All animals used in this research were sourced from Guangzhou KwangFeng Industrial Co., Ltd. (Guangzhou, China).

### 4.2. Experimental Animals

**Figure 7:**
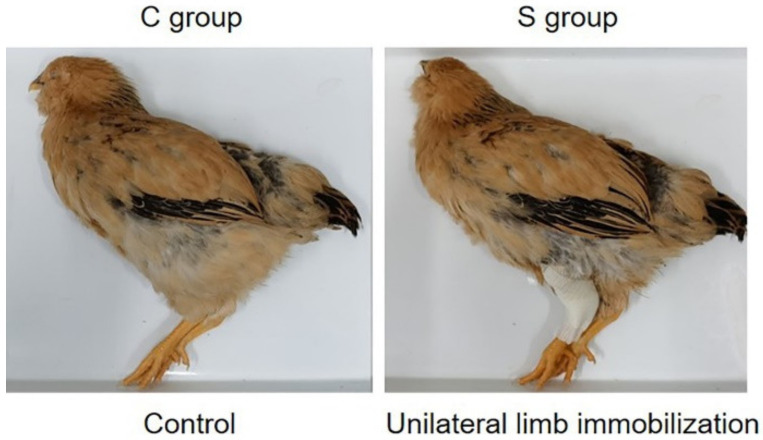
Schematic diagram of disuse muscle atrophy model.

Healthy and routinely immunized 49-day-old broilers (Sanhuang chicken, female, *n* = 12) with similar body weights were randomly divided into control group (C group, *n* = 6) and treatment group (S group, *n* = 6). The lower limbs of the C group (including the C-L group and C-R group) do not receive any treatment. In the S group, the tarsal joint and knee joint of the left lower limb (S-L group) were externally fixed with polymer fiber bandages, under which they could maintain a natural standing posture, and the muscle groups between the two joints could not complete the dynamic movement. Additionally, the right lower limb (S-R group) were performed as the C group. All broilers were reared in an environment with a light–dark cycle of 12 h and an ambient temperature of 24–26 °C for 2 weeks. The above operation and schematic diagram are listed in Table 1 and Figure 7.

### 4.3. Total RNA Extraction and RNA Sequencing

Total RNA was extracted by using Trizol reagent (Invitrogen, Carlsbad, CA, USA) following the manufacturer’s protocol. RNA integrity was detected in agarose gel electrophoresis, and RNA concentration and purity were determined by NanoDrop 2100 (Thermo Fisher Scientific, Fremont, CA, USA). BGI was responsible for RNA sequencing. About 50 to 150 ng of input RNA was withdrawn from each sample for RNA-seq library construction. The raw data obtained from sequencing were filtered to obtain clean data. The clean data were mapped to the reference genome (GRCg6a) by using HISAT [54]. The clean reads were aligned to reference sequences by using Bowtie2 [55]. The expression of genes or transcripts was calculated by RSEM [56]. The filtering conditions for the significantly differentially expressed transcripts were set as |fold change| ≥ 1 and Q-value ≤ 0.05. Cluster analysis for differentially expressed transcripts was performed by pheatmap 1.0.8. The raw data of RNA sequencing were submitted to the National Center for Biotechnology Information database (Accession Number: PRJNA777906).

### 4.4. RNA Extraction, cDNA Synthesis, and Quantitative Real-Time PCR (qRT-PCR)

RNA iso (TaKaRa, Otsu, Japan) was used to extract total RNA from muscles and cells according to the instructions. Reverse transcription to synthesize RNA cDNA was performed using HiScript Q-RT SuperMix for qPCR (+gDNA wiper) (Vazyme, Guangzhou, China). Using the ABI QuantStudio 5 instrument (Thermo Fisher Scientific, Fremont, NY, USA), the ChamQ Universal SYBR qPCR Master Mix (Vazyme) was used to perform qRT-PCR to detect the expression level of mRNAs. Data analysis of the results used the 2^−∆∆Ct^ method to calculate the relative expression of genes. The primers for real-time qPCR were designed by Premier Primer 5.0 software (Premier Biosoft, Palo Alto, CA, USA). Primers used for qPCR are listed in Table S5.

### 4.5. Cell Culture

The chicken primary myoblasts (CPMs) were isolated from leg muscle at the gestational age of 11 (Zhuhai Yuhe Co., Ltd., Zhuhai, China). First, the leg muscle tissues were cut into pieces after removing the skin and bone and then digested with pancreatin containing 0.25% EDTA for 15–20 min at 37 °C. Next, the digestion was terminated with Roswell Park Memorial Institute (RPMI)-1640 medium (Gibco, New York, NY, USA) containing 20% fetal bovine serum (FBS, Gibco), and the digested tissue fluid was filtered through a sterile filter. The single cells were collected by centrifugation at 1500 r/min for 5 min. Afterward, chicken primary myoblasts were obtained by the differential adhesion method. We cultured CPM cells in RPMI-1640 medium (Gibco, New York, NY, USA) with 20% FBS, 0.2% penicillin, and 0.2% streptomycin, then reduced FBS in the medium to 5% to induce differentiation.

### 4.6. Hematoxylin and Eosin Staining (HE)

The chicken gastrocnemius muscle tissues were taken, fixed with 4% paraformaldehyde, and embedded in paraffin. Tissue sections were stained with hematoxylin–eosin (HE), and then the images were analyzed using the morphological image analysis system provided by Jiangsu JEDA Science-Technology Development Company (Jiangsu, China).

### 4.7. Immunohistochemical Analysis

The chicken gastrocnemius muscle tissues were taken, fixed with 4% paraformaldehyde, and embedded in paraffin. Tissue sections were subjected to immunohistochemical analysis. Primary antibodies used for immunohistochemistry were mouse anti-myosin heavy chain, all fast isoforms (F-59, DSHB, 1:50 dilutions on paraffin sections), mouse anti-myosin heavy chain, and slow contracting muscle (S58, DSHB, 1:50 dilution on paraffin sections).

### 4.8. Western Blotting Assay

Ice-cold radio immunoprecipitation (RIPA) lysis buffer (Beyotime, Shanghai, China) with 1 mM phenylmethylsulfonyl fluoride (Beyotime, Shanghai, China) was used in the total protein extracted from gastrocnemius muscle tissues or CPM cells. SDS-page gel (10%) was used to separate proteins, and the separated proteins were transferred to the polyvinylidene fluoride (PVDF) membrane (Bio-Rad, Hercules, CA, USA). After 30 min of blocking, the membrane was incubated with anti-MYHC (1:200; B103, DSHB) or anti-GAPDH (1:2000; bsm-33033M, BIOSS) at 4 °C for 12 h. Anti-mouse secondary antibody (1:10,000; 7076P2, CST) was used to incubate the membranes. The ECL Peroxidase Color Development Kit (Beyotime, Shanghai, China) was used in chromogenic reactions by following the manufacturer’s protocol. Protein band visualization was performed using the Odyssey instrument (Li-cor, Lincoln, NE, USA).

### 4.9. Catalase (CAT) Activity Assay

The Catalase Assay Kit (Beyotime, Shanghai, China) was used to detect the CAT level by following the manufacturer’s protocol. The gastrocnemius tissue or cells were lysed with RAPI lysate, the supernatant was collected after centrifugation for determination, and then the absorbance at 520 nm was measured using a microplate reader (BioTek, Vermont, USA), and finally, the catalase activity in the sample was calculated.

### 4.10. Malondialdehyde (MDA) Content Assay

The Lipid Peroxidation MDA Assay Kit (Beyotime, Shanghai, China) was used to detect the MDA content by following the manufacturer’s protocol. The gastrocnemius tissue or cells were lysed with RAPI lysate, the supernatant was collected after centrifugation for determination, and then the absorbance at 532 nm was measured using a microplate reader (BioTek, Vermont, USA), and finally, the MDA content in the sample was calculated.

### 4.11. Total Antioxidant Capacity (T-AOC) Assay

The Total Antioxidant Capacity Assay Kit with a Rapid ABTS method (Beyotime, Shanghai, China) was used to detect the T-AOC by following the manufacturer’s protocol. The gastrocnemius tissue or cells were lysed with RAPI lysate, the supernatant was collected after centrifugation for determination, the absorbance at 414 nm was measured using a microplate reader (BioTek, Vermont, USA), and finally, the T-AOC in the sample was calculated.

### 4.12. Reactive Oxygen Species (ROS) Assay

The Reactive Oxygen Species Assay Kit (Beyotime, Shanghai, China) was used to detect the ROS level by following the manufacturer’s protocol. The cells were washed with PBS and incubated with DCFH-DA at 37 °C for 30 min. Then, the cells were further washed three times with PBS. The DMi8 fluorescence microscope (Leica, Heidelberg, Germany) was used to capture the random interfaces to visualize the ROS level. In addition, a microplate reader (BioTek, Winooski, VT, USA) was used to measure the fluorescence value at 480/528 nm.

### 4.13. Statistical Analysis

Based on at least three independent experiments, all experimental results are expressed as mean ± SEM. The statistical significance of the difference between the means was evaluated by performing an unpaired Student’s *t*-test. We considered *p* < 0.05 statistically significant. * *p* < 0.05; ** *p* < 0.01; *** *p* < 0.001.

## 5. Conclusions

In summary, this study constructed a broiler chicken disuse muscle atrophy model and analyzed differentially expressed genes in muscle atrophy by transcriptome sequencing. At the same time, it was proved by cellular experiments that excessive ROS can rapidly increase the level of oxidative stress in cells and lead to the atrophy of myotubes.

The results showed that, on the one hand, the method based on limb immobilization can successfully build a disuse muscle atrophy model, and then the sharply increased level of oxidative stress in the muscle tissue during this process is likely to be the “trigger” for inducing muscle atrophy. On the other hand, muscle catabolism in disuse muscle atrophy is carried out through the ATP–ubiquitin–proteasome proteolytic pathway.

## Figures and Tables

**Table 1:** Construction plan of disuse muscle atrophy model.

Control Group (C Group)		Treatment Group (S Group)	
Left lower limb	Right lower limb	Left lower limb	Right lower limb
(C-L group)	(C-R group)	(S-L group)	(S-R group)
No treatment	No treatment	Immobilization	No treatment

**Table 2:** Top 20 enriched KEGG pathways (*p* < 0.05).

Pathways	−log (*p* Value)	Rich Ratio	Focus Molecules
TGF-beta signaling pathway	3.67	0.13	ID1, ID2, ID3, SMAD6, SMAD7, BMP4, BMP5, FMOD, RBX1
FOXO signaling pathway	2.33	0.09	FBXO32, FOXO3, PLK2, PRKAB1, SGK1, HOMER3, GABARAPL1
Thyroid cancer	2.21	0.15	GADD45B, GADD45G, TPM3, CDKN1A, CCND1
Breast cancer	2.01	0.08	GADD45B, GADD45G, DLL4, CDKN1A, CCND1, NOTCH1
Glioma	2.00	0.10	GADD45B, GADD45G, CCND1, CDKN1A, PDGFB
Endometrial cancer	1.98	0.12	GADD45B, GADD45G, CCND1, CDKN1A, AXIN2
Small cell lung cancer	1.89	0.09	GADD45B, GADD45G, CCND1, CDKN1A, XIAP
Cardiac muscle contraction	1.86	0.11	MYH7B, MYH7, TNNC1, MYL3, MYH1B, ACTC1, TPM3
Notch signaling pathway	1.69	0.11	HES4, NOTCH1, LFNG, JAG1, DLL4
Calcium signaling pathway	1.66	0.07	ATP2A2, ATP2A3, ATP2B2, CAMK1D, CAMK4, GRIN2C
Nitrogen metabolism	1.66	0.11	HES4, NOTCH1, LFNG, JAG1, DLL4
Apelin signaling pathway	1.60	0.18	PRKAB1, APLNR, MYLK3
p53 signaling pathway	1.54	0.09	CDKN1A, CCND1, GADD45B, GADD45G, SESN1, AIFM2
Ribosome biogenesis in eukaryotes	1.51	0.09	RPP38, RPP30, HEATR1, NOP56, NAT10, UTP4
Pathways in cancer	1.44	0.05	GADD45B, GADD45G, XIAP, CDKN1A, CCND1, RBX1, ETS1
Other types of O-glycan biosynthesis	1.42	0.14	LFNG, ST6GAL2, EOGT
Hippo signaling pathway	1.41	0.07	WWC1, CCND1, FRMD6
Retinol metabolism	1.40	0.11	BCO1, ALDH1A2, CYP26A1, AOX1
cAMP signaling pathway	1.34	0.06	EDNRA, RELA, SOX9, PDE3A
PI3K-Akt signaling pathway	1.28	0.06	ANGPT4, PDGFB, F2R, KITLG, KDR, FLT1

## Data Availability

The raw data of RNA sequencing were submitted to the National Center for Biotech-nology Information database (Accession Number: PRJNA777906).

## Supplementary Materials

The following are available online at https://www.mdpi.com/article/10.3390/ijms23136892/s1.
