# Supplementary material for: Construction and Analysis of Disuse Atrophy Model of the Gastrocnemius Muscle in Chicken
Source: Int J Mol Sci. 2022 Jun 21;23(13):6892. doi: 10.3390/ijms23136892 (PMC9266690; doi:10.3390/ijms23136892)
Supplement: Supplementary file 1 [file ijms-23-06892-s001.zip › ijms-1752437-supplementary/Tables S1-S2, S5.pdf]

**Table S1.** The information of sequencing data.

| Sample | Total Raw Reads(M) | Total Clean Reads (M) | Total Clean Base (Gb) | GC (%) | Q30 (%) | Clean Reads Ratio (%) |
|---|---|---|---|---|---|---|
| C1-L | 50.83 | 45.89 | 6.88 | 50.66 | 87.25 | 90.28 |
| C1-R | 49.08 | 44.59 | 6.69 | 49.99 | 87.49 | 90.85 |
| C2-L | 50.83 | 46.36 | 6.95 | 51.25 | 87.38 | 91.2 |
| C2-R | 49.08 | 44.93 | 6.74 | 50.52 | 87.35 | 91.54 |
| C3-L | 49.08 | 45.1 | 6.77 | 50.40 | 88.09 | 91.89 |
| C3-R | 52.59 | 48.02 | 7.2 | 49.91 | 87.54 | 91.31 |
| S1-L | 52.43 | 47.34 | 7.1 | 50.66 | 87.47 | 90.28 |
| S1-R | 49.08 | 45.42 | 6.81 | 49.54 | 87.66 | 92.55 |
| S2-L | 49.08 | 44.73 | 6.71 | 51.04 | 87.88 | 91.14 |
| S2-R | 49.08 | 44.74 | 6.71 | 50.28 | 87.48 | 91.15 |
| S3-L | 50.83 | 46.17 | 6.93 | 50.04 | 87.56 | 90.83 |
| S3-R | 49.08 | 44.54 | 6.68 | 50.86 | 86.87 | 90.76 |
| S4-L | 49.74 | 45.09 | 6.76 | 51.39 | 87.03 | 90.65 |
| S4-R | 49.08 | 44.88 | 6.73 | 50.44 | 87.5 | 91.45 |

**Table S2.** Genome alignment statistical of clean reads.

| Sample | Total Mapping Clean Reads(M) | Total Mapping Ratio(%) | Uniquely Mapping Ratio(%) |
|---|---|---|---|
| C1-L | 45.89 | 84.56 | 79.77 |
| C1-R | 44.59 | 85.28 | 81.05 |
| C2-L | 46.36 | 85.19 | 81.04 |
| C2-R | 44.93 | 85.13 | 80.41 |
| C3-L | 45.10 | 85.62 | 80.93 |
| C3-R | 48.02 | 86.71 | 83.60 |
| S1-L | 47.34 | 83.96 | 80.51 |
| S1-R | 45.42 | 87.15 | 83.21 |
| S2-L | 44.73 | 84.19 | 78.44 |
| S2-R | 44.74 | 85.32 | 80.48 |
| S3-L | 46.17 | 86.91 | 83.66 |
| S3-R | 44.54 | 84.15 | 79.06 |
| S4-L | 45.09 | 83.67 | 78.46 |
| S4-R | 44.88 | 84.76 | 79.62 |

**Table S5.** Primers used for qPCR

| Fragment name | Fragment sequences (5’ to 3’) | Size (bp) |
|---|---|---|
| CCNB2 F | CAGTAAAGGCTACGAAAG | 133 |
| CCNB2 R | ACATCCATAGGGACAGG | |
| CCND1 F | CAGAAGTGCGAAGAGGAAGT | 188 |
| CCND1 R | CTGATGGAGTTGTCGGTGTA | |
| CCND2 F | AACTTGCTCTACGACGACC | 150 |
| CCND2 R | TTCACAGACCTCCAACATC | |
| PCNA F | GTGCTGGGACCTGGGTT | 217 |
| PCNA R | CGTATCCGCATTGTCTTCT | |

| | | |
|---|---|---|
| P21 F | GAAGAGTTGTCCACGATAAGC | 247 |
| P21 R | TTCCAGTCCTCCTCAGTCC | |
| CDKN1A F | CAGCAGCAAAGCGTGCAGG | 121 |
| CDKN1A R | CTCGGTCTCGAAGTTGAAGTTCC | |
| CDKN1B F | GCTGTGCTGGGCTGAA | 207 |
| CDKN1B R | GGACGAAAGGATGTGGG | |
| CASP3 F | TGGCCCTCTTGAACTGAAAG | 139 |
| CASP3 R | TCCACTGTCTGCTTCAATACC | |
| CASP9 F | TCCCGGGCTGTTTCAACTT | 207 |
| CASP9 R | CCTCATCTTGCAGCTTGTGC | |
| MYHC F | CTCCTCACGCTTTGGTAA | 213 |
| MYHC R | TGATAGTCGTATGGGTTGGT | |
| MYH1A F | GAGTCTGTCTCCAAAGCCA | 154 |
| MYH1A R | ATTCACCTGATTCTGTCTGC | |
| TNNC2 F | CCAGATGAAAGAGGACGCCAAG | 233 |
| TNNC2 R | GCACACCCTCCATCATCTTCAG | |
| TNNI2 F | GATGAGGAAAGGTATGACACAGA | 214 |
| TNNI2 R | CCTTCTTGACTTGCTTCAGGTT | |
| TNNT3 F | GAAAGAGCAGAGCAACAGAGAAT | 205 |
| TNNT3 R | TTTGCTTCTTCCCTCTCTTCTG | |
| MYH7B F | TACCTATTCGGGGCTCTTCT | 175 |
| MYH7B R | GGACTGGTTCTCACGATTGC | |
| TNNC1 F | GAGGTGGATGAGGATGGCAG | 199 |
| TNNC1 R | GATCGTCTCTCCAGTTGCCTG | |
| TNNI1 F | ACCTGAAGCTCAAAGTGCTCGA | 212 |
| TNNI1 R | CTCCACGTTCTTGCGCCAATCA | |
| TNNT1 F | CCGAGGATGACGCCAAGAAGAA | 252 |
| TNNT1 R | GCAGCTTCTCCATCAGGTCGAA | |
| FOXO1 F | TACTTCATGTCTAAATCGCTTGTCG | 127 |
| FOXO1 R | CACCCTTCCGTAGCCGTTG | |
| FOXO3 F | GTGCGGTGCGTGCCCTACTT | 132 |
| FOXO3 R | GCTCTTCCCAGTGCCTTGGT | |
| MuRF1 F | GGCAGCAGCATCATCTCGG | 224 |
| MuRF1 R | CCTCGCAGGTGACGCAGTAG | |
| Atrogin1 F | TCAACGGGTCGGCAAGTCT | 192 |
| Atrogin1 R | TCCCTCCCATCGCTCAGTC | |
| APOD F | CCGCTTCAACTGGTTTATGC | 177 |
| APOD R | ATGCTCTTCAGGTGCTCTACA | |
| UCP3 F | ATCGCCAGGGAGGAGGGAGT | 170 |
| UCP3 R | AAAGCAGCCACGAAGTGACAGG | |
| β-actin F | GATATTGCTGCGCTCGTTG | 195 |
| β-actin R | TTCAGGGTCAGGATACCTCTTT | |
